# The predictive validity of the family risk survey and child risk survey for identifying persistent firesetting risk

**DOI:** 10.1002/jclp.23435

**Published:** 2022-08-26

**Authors:** Kara Dadswell, Stjepan Sambol, Dorothy Bruck, Michelle Ball

**Affiliations:** ^1^ Institute for Health and Sport Victoria University Footscray Park Victoria Australia; ^2^ College of Health and Biomedicine, Discipline of Psychology Victoria University Melbourne Victoria Australia

**Keywords:** child and adolescent mental health, screening

## Abstract

Young firesetter behavior poses significant risks to individuals and communities. Intervention is important to mitigate youth firesetting, and treatment needs vary depending on underlying motives. Effective screening of persistent firesetter risk to inform intervention approach is critical to ensure appropriate matching of risk and needs. This study aimed to evaluate the utility of the child risk survey (CRS) and family risk survey (FRS) for predicting persistent firesetting risk, and subsequent triaging of cases toward the appropriate treatment. A total of 61 families engaged with the Firelighting Consequences Awareness Program, Melbourne, Australia, completed the CRS and FRS preintervention, and reported their firesetting behavior 1‐year postintervention. The CRS was not effective for correctly predicting persistent and nonpersistent firesetters. The FRS was successful at predicting persistent firesetters 85% of the time, but had a high rate of false positives, overclassifying nonpersistent firesetters as high risk. Finally, the actual rate of firesetters that would be deemed suitable for each of the three recommended interventions based on the CRS and FRS scoring protocols was substantially different to the expected rates described in the accompanying manual. Implications for service provision are discussed.

## 1 INTRODUCTION

Young firesetter behavior is a global issue with financial and human costs and consequences. It is also associated with adult arson (Ducat & Ogloff, 2011) and other antisocial behaviors (Lambie et al., 2013). Clear prevalence rates for firesetting in young people (under 18) are not available. The covert nature of firesetting behavior, combined with variations in how firesetting behavior is defined and measured across the literature, has produced an inconsistent picture of the problem (Sambrooks et al., 2021). Nonetheless, figures do highlight that a problem exists. For example, in 2014, young people were the perpetrators of deliberate firesetting in 34% of cases in the US where charges were laid (Campbell, 2017). In an Australian sample, 67% of offending, and 38% of nonoffending youth admitted to lighting an object on fire at least once (Watt et al., 2015).

Several motivations that underpin young firesetter behavior have been postulated in the research literature. Fineman (1995) presented eight broad firesetter typologies described by their dominant firesetting drive—four of which are most commonly associated with youth firesetters and aligned to treatment options: (1) the curious type; (2) the “cry for help” type; (3) the delinquent type; and (4) the severely disturbed type. These typologies vary in degree of underlying psychopathology and risk of persistent and problematic firesetting, with the curious type considered “nonpathological,” and the latter types “pathological.” An understanding of motivating factors is central to effectively addressing the core of the issue. To reduce the risk of continued firesetting and progression of criminal activity in general, young firesetters of any kind should be engaged in intervention that meets their specific needs (Perks et al., 2019). While young firesetters are a heterogeneous group, and sometimes engage in firesetting for more than one reason (Walsh & Lambie, 2013), classification systems like the one proposed by Fineman may be useful for informing treatment needs. Intervention and treatment programs for young firesetter behavior generally include either fire safety education, mental health services, or a combination of both. The Federal Emergency Management Agency, United States Fire Administration (FEMA; 2002) estimates 60%–70% of young people who light fires would be considered low risk and fire safety education intervention is believed enough to prevent any future firesetter activity. However, this means that an estimated 30%–40% of firesetters are assumed to present increased risk and experience significant psychosocial issues that may contribute to persistent firesetter behavior if treatment does not target the root of the problem (Dadswell et al., 2021; FEMA, 2002).

The risk‐need‐responsivity (RNR) model is the most empirically supported and prominent offender rehabilitation framework used globally to guide psychological assessment and treatment in forensic settings (Andrews & Bonta, 2010). Tenants of the RNR model suggest that: (1) the type and degree of intervention service should be dependent on identified recidivism risk level; (2) treatment must target the needs of individuals specifically according to their static and dynamic risk factors; and (3) treatment delivery must respond to individual differences in cognitive ability, preferred learning style, cultural backgrounds and any other factors that could impact on the success of intervention (Andrew & Bonta, 2010; Fritzon et al., 2021). As firesetters are such a heterogeneous group all firesetter interventions would be strengthened by adopting the RNR framework into processes for identifying risk and associated need (Foster, 2020). The most utilized model of intervention targeted at known firesetters is fire safety education provided by fire service professionals (Dadswell et al., 2021; FEMA, 2002; Foster, 2020; Haines et al., 2006; Johnston & Tyler, 2022; Palmer et al., 2005). Generally, fire services deploy specifically trained firefighters to provide these educational services to young people. The content of intervention commonly revolves around teaching fire safety skills, the dangers of firesetting, practical fire knowledge, competency around fire recognition, how to respond in an emergency, and safe fire use (Palmer et al., 2007). This type of secondary (reactive) intervention may also include some basic social and behavioral modification techniques (Muller & Stebbins, 2007). Fire safety education has shown to be effective for nonpathological firesetters, however, mental health services are suggested as necessary for young firesetters with what are considered more pathological motivations (Dadswell et al., 2021; FEMA, 2002; Johnston & Tyler, 2022). Very few integrated fire safety education and mental health service programs exists that target young firesetter behavior. Programs targeting young firesetters generally use a disconnected single agency approach to intervention, sometimes completing risk assessments to refer firesetters to separate, relevant services (Henderson et al., 2010), but this does not occur in all programs.

Fire service‐led intervention programs can often be a first‐point of professional contact for young people with problematic underlying psychosocial disturbances when firesetting behavior is identified (Henderson et al., 2010; Lambie et al., 2019). Although fire safety education delivered by fire services is viewed as appropriate for low‐risk firesetters, a review of interventions conducted by Baretto et al. (2004) reported that evidence suggests fire safety education alone has limited effectiveness for eradicating firesetting behavior in young people marked by significant behavioral or emotional disturbance. As previously described, approximately one‐third of young firesetters are contemporaneously experiencing psychosocial dysfunction and would benefit from mental health support (Dadswell et al., 2021). Despite their limitations for treating pathological firesetters, fire service‐led fire safety education programs have capacity to screen for persistent firesetting risk and potential psychosocial concerns and provide mental health referral. Therefore, programs of this style, and the families they serve, would benefit from an effective early triaging process to correctly identify and filter firesetters who are at risk of repeated misuse of fire behavior, and whom have increased psychosocial concerns to appropriate services (Lambie et al., 2019). It is expected that future firesetting behavior would be reduced through referral to, and subsequent uptake of, mental health services that are correctly tailored to the needs of each individual case.

## 2 THE JUVENILE FIRESETTER CHILD AND FAMILY RISK SURVEYS

The US Fire Administration's National Fire Academy offers a Youth Firesetting Prevention and Intervention course for youth firesetting program personnel. Included in the course materials are a number of screening tools (e.g., The Child and Parent Interview designed by Fire Stoppers Children's Fire Prevention Program of Washington, the FEMA Comprehensive Fire Risk Long Form) intended for use by fire service‐led firesetter intervention programs to guide intervention choices. However, most are theoretically driven, or have not been psychometrically evaluated. The brief Juvenile firesetter child and family risk surveys (CRS and FRS) were chosen for use in this study because they have statistical underpinnings, parsimony, and on face value appear practical for use. The CRS and FRS were developed by Moynihan and Flesher (1998) after it was determined that fire services needed a parsimonious method for assessing fire risk to inform appropriate intervention (DiMillo, 2002). The CRS and FRS were developed using a thorough statistical process that consisted of factor and correlation analyses to reduce the number of items, followed by regression techniques to establish numerical weightings associated with each question, and the derivation of appropriate cut‐off values to classify risk. Although the development of these tools occurred some 20+ years ago, and there has been some progression in our understanding of firesetting behavior since this time, these tools are still widely used in fire service‐led firesetter intervention programs (e.g., Sacramento Valley [California] Juvenile Firesetter Program; University of Michigan Trauma Burn Center's Straight Talk Youth Fire Prevention and Intervention Program) and warrant validation.

The basic premise of CRS and FRS is that the total scores on the surveys correspond to one of three risk categories (little, definite, and extreme) reflecting the likelihood of persistent firesetting and the severity of firesetting behavior (FEMA, 2002) to inform intervention. The risk categories align to Fineman's (1995) typologies described above, and each risk category has a recommended intervention attached. The little risk category captures naively curious firesetters, and fire safety education is the recommended intervention. As previously stated, the majority (60%–70%) of young firesetters are expected to fit into this category. The second most common classification is the definite risk category, encompassing an anticipated 30%–40% of firesetters. The definite risk category includes young people with emotional (i.e., “cry for help” type) and behavioral (i.e., delinquent type) disturbances. A combination of fire safety education and mental health intervention is recommended for anyone screened as definite risk. Last, the extreme risk category captures severely disturbed firesetters, and standalone mental health treatment is advised as the *only* suitable intervention. However, these young firesetters are considered rare with an estimated 1%–2% of young firesetters falling into this category (Fineman, 1995; FEMA, 2002). Therefore, fire safety education is considered a critical component of intervention for almost all cases (little and definite risk).

Despite being developed more than 20 years ago, the CRS and FRS are yet to have their psychometric properties evaluated, and their predictive capability determined empirically. Notwithstanding, the FEMA tools have provided fire services in the USA with a tangible measure that allows fire service personnel to attend to the issue of firesetter assessment, in absence of other options (MacKay et al., 2012). Further empirical investigation into their utility is long overdue. Preliminary findings reported in another publication by the authors (Dadswell et al., 2021) indicated that the FRS has some utility for distinguishing between persistent and nonpersistent firesetters, with persistent firesetters scoring significantly higher on this tool, however, the CRS scores did not differ between persistent and nonpersistent firesetters.

Therefore, the overall aim of this project was to extend the preliminary evaluation of the CRS and FRS (Dadswell et al., 2021), and to assess the predictive validity through identifying their effectiveness for predicting persistent firesetting risk, and subsequent triaging of cases toward the appropriate treatment approach. Specifically, the ability of the FEMA tools to accurately predict and classify persistent and nonpersistent firesetters as high and low risk at the time of initial intervention contact was assessed.

## 3 METHOD

### 3.1 Participants

As part of a larger study, participants were recruited through their engagement with the Firelighting Consequences Awareness Program (Fire‐CAP) in Victoria, Australia and data were collected pre‐ and postintervention. The Fire‐CAP consists of operational firefighters trained specifically as Fire‐CAP practitioners to deliver targeted fire safety education to young people who have been referred for engaging in firesetting behavior. Referrals come from an array of sources including local fire services, parents/carers, schools, police, or juvenile justice, and firesetting incidents vary widely in severity. The Fire‐CAP practitioners visit the young people and their parents/guardians in their homes to deliver the program using resources appropriate to different ages and developmental stages (e.g., videos, handouts).

Data were collected between December 2010 and April 2013, and during this 2.5‐year period, 345 cases were actioned by the Fire‐CAP. Of these, a total of 169 cases initially agreed to participate, with 61 families completing and returning the pre‐ and postintervention questionnaires. The final sample consisted of young people including 55 males, and six females, aged between 5 and 17 years (*M* = 10.9, *SD* = 3.3). A total of 32.7% of cases in the sample were reported as persistent firesetters (including matchplay, firesetting, or both) within the 12 months period post‐Fire‐CAP intervention; 67.3% were nonpersistent firesetters. Varied types and degree of community mental health treatment, including psychologists and school counselors was accessed by 60% of persistent firesetters of their own accord during this time compared to 22% of nonpersistent firesetters (note: 30% of persistent firesetters and 54% of nonpersistent firesetters did not seek any mental health treatment between intervention and follow‐up. A further 10% and 24% of each group respectively did not provide any information regarding mental health treatment usage).

## 4 MATERIALS

### 4.1 Preintervention

#### 4.1.1 The juvenile firesetter child and family risk surveys

The CRS is a tool for fire service‐led firesetter interventions and is administered directly to each young firesetter by an intervention practitioner (Moynihan & Flesher, 1998). The CRS contains 14 questions relating to the child or adolescent's interpretation of their family dynamics (e.g., Do you see your father as much as you'd like?), general behavior (e.g., When you're asked to do something do you usually do it?), history of firesetting (e.g., Besides this fireplay or firesetting incident, how many other times have you played with fire, including matches or lighters, or set something on fire?), most recent fire incident (e.g., Did you intend to play with the fire or set the fire, i.e., did you play with or set the fire on purpose?) and specific fire interest (e.g., Do you like to look at fire for long periods of time?). In contrast, the FRS is designed for the parent/carer to complete. It contains seven questions relating to the child's curiosity about fire (e.g., If you had to describe your child's curiosity about fire, would you say it was absent, mild, moderate, or extreme?), impulsive conditions (e.g., Has your child been diagnosed with any impulse control conditions, such as Attention Deficit Disorder or Attention Deficit Disorder with Hyperactivity?), antisocial behaviors (e.g., Has your child ever stolen or shoplifted?) and history of firesetting (e.g., Besides this fireplay or firesetting incident, how many other times has your child played with fire, including matches or lighters, or set something on fire?).

The CRS and FRS are intended to be administered to parents/carers and children synonymously. Possible responses to the questions on both tools are categorical and have corresponding numerical weightings based on their degree of association with persistent firesetting. The quantitative responses from both surveys are scored separately, and the young person is classified according to their risk of persistent firesetting on each (e.g., little, definite, or extreme risk). Children categorized as being at little risk on both surveys (FRS scores < 429, CRS scores < 511) are deemed suitable for fire safety education intervention. Young people classified as definite risk on at least one of the surveys (FRS scores 429–457, CRS scores 511–540), are given fire safety education and a mental health referral. Those found to be at extreme risk on either or both surveys (FRS scores > 457, CRS scores > 540) are considered ineligible for fire safety education and are referred on for mental health intervention only. The regression techniques used by Moynihan and Flesher (1998) to develop the CRS and FRS demonstrated predictive validity as some included items were significant predictors of juvenile firesetting. It should be noted that some nonsignificant (*p* = 0.05) items were retained because they added further explanatory power to the overall model. In the current study, the total scores on the FRS and CRS showed a strong relationship (*r* = 0.520), suggesting both measures assessed similar constructs. As the reliability and validity of these measures was yet to be formally assessed, no referrals to mental health services were made on the basis of these scores in this study (note: as abovementioned in the participants section, some participants sought mental health treatment post‐Fire‐CAP of their own accord).

### 4.2 Stage two: Postintervention

#### 4.2.1 The fire history screen (FHS)

The FHS (Kolko & Kazdin, 1988) is a measure of firesetting behavior. The 13‐item tool was adapted for this study to assess persistent firesetting/matchplay. The questionnaire elicits information relating to recent firesetting behavior (within the past 12 months post‐Fire‐CAP intervention), including matchplay and firesetting incidents. The severity and frequency of the incidents are captured. The FHS has demonstrated adequate psychometric properties, with moderate–high test–retest reliability over a 2‐week period (0.43−0.86), and no significant difference between post‐intervention scores and a 1‐year follow‐up. Furthermore, responses from recidivist firesetters added significant incremental variance in predicting follow‐up firesetting (Kolko, 2001; Kolko et al., 2001). For the current study, this tool was exclusively used to categorize participants as persistent or nonpersistent firesetters (similar to methods used by Dadds & Fraser, 2006). If participant responses indicated matchplay and/or firesetting on either of both of the following two items: “Did your child ever just play with matches, lighters, or the stove without burning anything else in the last 12 months” or “in the last 12 months how many times did your child burn something like paper, clothes, furniture, walls, or the house, or set something on fire, without permission from an adult excluding the incident for which the child adolescent was referred to the juvenile fire awareness program” they were classified as persistent firesetters. If these responses indicated no engagement with matchplay and firesetting in the past 12 months they were classified as nonpersistent firesetters.

## 5 PROCEDURE

### 5.1 Ethics approval

Approval from the Victoria University Human Research Ethics Committee was granted for this project.

### 5.2 Data collection: Preintervention

As part of a larger study, the Fire‐CAP state coordinator directly contacted all parents/carers of cases referred to the program across a 2.5‐year period to inform them about the research. For the larger study, consenting parents/carers completed a battery of assessment measures including the FRS. At the initial Fire‐CAP interview session before the intervention commencement, the CRS was administered to children or adolescents of consenting parents/carers by the Fire‐CAP practitioner. The questions were read aloud to each young person, and they responded in the presence of their parent/carer.

### 5.3 Data collection: Postintervention

In the 12th month after the Fire‐CAP intervention sessions had concluded, participants completed the FHS as part of the follow‐up assessment battery for the larger study.

### 5.4 Data analysis

All statistical analyses were conducted using the Statistical Package for Social Sciences, Version 22 (SPSS). Descriptive statistics were used to assess the percentage of little, definite, and extreme risk firesetters according to the CRS and FRS classification scores. For the purpose of conducting 2 × 2 cross‐tabulations, participants classified as definite or extreme risk screens on the FRS and/or CRS were merged into a single category (i.e., high risk). The little risk screens were reclassified as low risk and compared with the new high‐risk category. First, two chi‐squared tests of independence were conducted to investigate the difference in CRS and FRS screens between persistent and nonpersistent firesetters. Second, odds ratios (ORs) were calculated to determine the probability of persistent firesetters obtaining high‐risk screens on the CRS and FRS compared to the probability of nonpersistent firesetters obtaining high‐risk screens.

The predictive validity of the CRS and FRS was investigated with six statistics (see Supporting Information: Appendix for formulae): (1) sensitivity provides a measure of true positives, the extent to which CRS and FRS high risk screens can accurately predict persistent firesetters; (2) specificity reflects true negatives, the extent to which CRS and FRS low risk screens can accurately predict nonpersistent firesetters; (3) positive predictive value (PPV) is the probability of high risk screens accurately identifying persistent firesetters; (4) negative predictive value (NPV) is the probability of low risk screens accurately identifying nonpersistent firesetters; (5) positive likelihood ratio (PLR) is the likelihood of a high risk screen correctly identifying a persistent firesetter compared to the likelihood of a nonpersistent firesetter being incorrectly labeled as high risk; and last, (6) negative likelihood ratio (NLR) is the likelihood that a persistent firesetter would be incorrectly screened as low risk compared to the likelihood of a low risk screen correctly identifying nonpersistent firesetters (Lalkhen & McCluskey, 2008; Shreffler & Huecker, 2021).

## 6 RESULTS

### 6.1 Chi‐square test of independence and OR

Table 1 displays the observed frequency, expected frequency, and the overall screening accuracy of the FRS and CRS. The results of the chi‐square tests of independence demonstrated a significant difference between persistent and nonpersistent firesetters on FRS screens *χ* ^2^ (1, *n* = 60) = 7.813, *p* = 0.005, *φ* = 0.361, however no significant difference was observed on CRS screens *χ* ^2^ (1, *n* = 59) = 1.936, *p* = 0.164, *φ* = 0.181. These findings suggest the distribution of high‐risk and low‐risk screens, from the CRS, across persistent and nonpersistent firesetters were roughly equivalent. In contrast, the FRS showed significantly fewer than expected low‐risk screens for persistent firesetters, and fewer high‐risk screens for nonpersistent firesetters.

**Table 1:** Observed and expected frequencies, and screening accuracy of the family risk survey (FRS) and child risk survey (CRS)

FRS result	Persistent firesetter	Nonpersistent firesetter	Total
High risk	TP	FP	36
Observed	17 (28.33%)	19 (31.67%)	
Expected	12	24	
Low risk	FN	TN	24
Observed	3 (5%)	21 (35%)	
Expected	8	16	
Totals	20	40	60

CRS result	Persistent firesetter	Nonpersistent firesetter	Total
High risk	TP	FP	28
Observed	11 (18.64%)	17 (28.81%)	
Expected	8.5	19.5	
Low risk	FN	TN	31
Observed	7 (11.86%)	24 (40.68%)	
Expected	9.5	21.5	
Total	18	41	59

The OR for firesetter classification receiving a high‐risk screen on the FRS was 6.26, 95% CI [1.58, 24.78], and on the CRS was 2.22, 95% CI [0.714, 6.89]. This indicates that the likelihood of persistent firesetters being identified as high risk on the FRS was 6.26 times more likely than nonpersistent firesetters being identified as high risk. Conversely, on the CRS persistent firesetters are 2.22 times more likely to be identified as high risk compared to nonpersistent firesetters. Therefore the FRS was considerably more likely to classify persistent firesetters as high risk compared to the CRS.

### 6.2 Predictive validity of the juvenile firesetter child and family risk surveys

The FRS demonstrated a sensitivity rate of 85% and specificity rate of 52.5%. This indicates high‐risk screens correctly identified persistent firesetters 85% of the time, and the low‐risk FRS screens predicted a nonpersistent firesetter 52.5% of the time. The PPV was 47.2% and the NPV was 87.5%. The low PPV indicates that incorrect high‐risk screens for nonpersistent firesetters are frequent (52.8% of the time) with the FRS. Conversely, the high NPV indicates that incorrect low‐risk screens for persistent firesetters are minimal (12.5% of the time). The PLR was 1.79:1, and the NLR was 0.28:1. The PLR value suggests only a small increase in the probability (1.79 times more likely) of a high‐risk screen correctly identifying a persistent firesetter on the FRS compared to a nonpersistent firesetter being incorrectly labeled as high risk. The NLR value indicates that there is a low likelihood that a persistent firesetter would be incorrectly screened as low risk compared to the likelihood of a low‐risk screen correctly identifying a nonpersistent firesetters. Specifically, the probability of having a low‐risk screen for a persistent firesetter is about one‐quarter of that of nonpersistent firesetters.

The CRS accurately predicted just over half of all persistent and nonpersistent firesetters. The high‐risk screens only correctly identified 61.1% of persistent firelighters (sensitivity), and the low‐risk screens only correctly identified 58.5% of nonpersistent firesetters (specificity). The PPV was 39.3% and the NPV was 77.4%. The low PPV indicates that incorrect high‐risk screens for nonpersistent firesetters are frequent (61.7%) with the CRS. The high NPV indicates that incorrect low‐risk screens for persistent firesetters occur about one‐quarter of the time (22.6%). The PLR was 1.47:1, and the NLR was 0.66:1. The PLR value suggests a very small increase in the probability (1.47 times more likely) of a high‐risk screen correctly identifying a persistent firesetter on the CRS compared to a nonpersistent firesetter being incorrectly labeled as high risk. The NLR value indicates that there is a moderate likelihood that a persistent firesetter would be incorrectly screened as low risk compared to the likelihood of a low‐risk screen correctly identifying nonpersistent firesetters. Specifically, the probability of having a low‐risk screen for a persistent firesetter is about two‐thirds of that of nonpersistent firesetters.

### 6.3 Classification accuracy

Based on the FEMA (2002) manual risk categorization protocols, 21 firesetter cases (34.4%) would have been considered low risk and given the fire safety education intervention. A further two cases would have been screened as definite risk (3.3%) and considered suitable to receive fire safety education and a mental health referral. A total of 38 cases (62.3%) would have been classified in the extreme risk category and ineligible for fire safety education intervention. Instead, a mental health referral would have been provided to these extreme risk cases.

## 7 DISCUSSION

The overall aim of this study was to assess the predictive validity of the FEMA instruments, specifically the CRS and FRS, by examining their ability to correctly identify persistent firesetting risk, and to successfully triage cases toward recommended treatment methods. One‐third of the study sample reported persistent firesetter behavior 12 months after engagement with a Fire‐CAP. It therefore seems that fire safety education intervention as the sole treatment is effective for most (two‐thirds) young people who engage in firesetting behavior, but not sufficient to eradicate this behavior for all participants. The rate of persistent firesetting is consistent with a recent meta‐analysis investigating firesetter reoffending (Perks et al., 2019), but higher than the rare studies reporting evaluations of firesetter interventions specifically (e.g., Lambie et al., 2019; 5% arson recidivism). These discrepancies in persistent firesetting figures are likely to be a result of the varied measurement of persistent firesetting used as an indication of program success (e.g., criminal convictions of fire offenses verses parent‐report firesetting and/or matchplay).

When assessing the predictive validity of the CRS and FRS and their utility for identifying future persistent and nonpersistent firesetters, findings were mixed. On face value, preliminary analyses showed the FRS to have some utility for predicting risk, but the same was not found for the CRS. Specifically, those screened as high risk using the FRS were more likely to become persistent firesetters than those screened as low risk. In addition, nonpersistent firesetters were less likely to screen as high risk and more likely screen as low risk than persistent firesetters. When investigating the CRS, the distribution of persistent and nonpersistent firesetters across low and high‐risk screens were equal, suggesting it was unable to distinguish those who continued firesetting from those who did not. However, the preliminary analyses do not paint the full picture. Further investigation of validity of both tools revealed the CRS demonstrated virtually no ability to correctly identify both persistent and nonpersistent firesetters, while the FRS was successful at predicting a persistent firesetters 85% of the time, demonstrating appropriate sensitivity. Though, the FRS also demonstrated a high rate of false positives, overclassifying half of the cases of nonpersistent firesetters as high risk when these participants did not actually light any further fires. In a sense, it appears the FRS has a high rate of persistent firesetter detection because it flags the majority of screened cases as high risk.

It is important that validity measures of screening tools are considered in the context in which the tools will be used (Bartol, 2015). Admittedly in this scenario it is more troublesome for a screening tool to fail to identify an at‐risk case than to over‐classify and predict high risk incorrectly, but over‐classification can also be associated with problems. For example, unnecessary stigmatization of the young person, financial impost arising from referral to mental health services, or detrimental exclusion from fire safety education may result from inaccurate risk classification. Essentially, while in this context the most important consideration is for the tool to detect high‐risk cases effectively—too much over classification may make the screening tool void of its purpose, and no more effective than giving all cases a mental health service referral as standard practice, or potentially harmful, if cases are unnecessarily denied access to fire safety education.

Interestingly, on closer inspection of the risk categorization of the young firesetters, some concerns arose. If the CRS and FRS were implemented into the Fire‐CAP and protocols according to the FEMA manual (FEMA, 2002) were followed, only 37.7% of the Fire‐CAP clientele would have been provided with the fire safety education program. The remaining 63.3% fell into the extreme risk category and would have been considered ineligible for fire safety education and referred on to mental health services alone. Only two cases would have been given the combined treatment approach of fire safety education and mental health referral. These findings are in contrast to the frequency expectations reported in the FEMA manual, where only 1%–2% of young firesetters are expected to fall into the extreme risk category. The largest portion of cases is expected to be seen in the low‐risk category, and about a third in the definite risk category, which are all still eligible to receive the fire safety education program (plus mental health referral for definite risk). It is suspected that majority of the 63% of cases screened as extreme risk have been incorrectly classified and these tools are exaggerating the level of risk (e.g., these cases are more likely to be “definite” risk cases rather than “extreme”). This is a concern because this higher risk categorization than necessary, renders them ineligible for fire education support. Moreover, fire safety education intervention that plays a crucial role in the eradication of firesetting behavior (MacKay et al., 2012), and should be a component of treatment for the large majority of identified firesetters.

A possible explanation for the distribution of classifications seen here lies in the score weightings assigned to some item responses. For example, the parent‐report FRS item “Besides this fireplay or firesetting incident, how many others times has he/she played with fire, including matches or lighters, or set something on fire?” has the response options 1, 2, 3–4, and 6+. The corresponding score contributing to the FRS total for each response option is 89, 168, 336, and 504 points, respectively. Therefore, if a parent reports that their child has played with fire six or more times in the past, 504 points will contribute to their FRS total which would automatically push them above the 457 point threshold, deeming them ineligible for fire safety education and referred on for mental health intervention only. This is a concern, as fire safety education may be a really important component of treatment for a child with this history. Future research should more closely examine the scoring system for the FEMA tools (e.g., the weight of items contributing to the overall score) to ensure individuals who may benefit from fire safety education are not excluded. Despite the prolific use of the CRS and FRS some 20 years after their development, justification for their scoring systems has never been examined in a peer‐reviewed publication.

Consistent with the RNR framework (Andrews & Bonta, 2010; Fritzon et al., 2021), risk identification is the first step in any offender rehabilitation program before targeting of specific needs occurs in intervention. The literature and the FEMA tools alike anticipate that a degree of psychopathology is present for about 30%–40% of firesetters presenting to fire safety education programs, and it is probable that the persistent firesetters identified in this study require some psychological intervention. Even some of the nonpersistent firesetters displayed markers indicative of mental health service need. As fire service‐led youth firesetter intervention programs are often a first point of professional contact, they are well placed to link young people with mental health services (McCarty & McMahon, 2005). Therefore, an effective screening process within standalone education programs is imperative. This would enable more high‐risk cases, and those with mental health concerns, to be objectively identified by fire service practitioners, and filtered through to separate mental health service providers. A valid and reliable screening process would provide a cost‐effective and simple solution to aid in attending to the multifaceted issue of firesetting, where costly multidisciplinary programs are not feasible. While the FRS has demonstrated to be useful to some extent in achieving this, future research should look to improve the predictive accuracy of a screening tool for fire service‐led firesetter intervention programs. In particular, the tendency for the tool to potentially over classify cases as extreme risk, when this precludes them from accessing fire safety education should be of paramount concern.

## 8 LIMITATIONS

This study is not without limitations. A low rate of participation in the research (17.6%) from the total population pool (*N* = 346) limits the findings of this study. Due to the methodological challenges of conducting research with vulnerable groups (Horowitz et al., 2002), it is anticipated that some of the highest risk young people who accessed the Fire‐CAP did not participate in this study. This possible bias in recruitment suggests that the persistent firesetting rate found in this study may under‐represent the problem of ongoing firesetting in this cohort. Additionally, 90% of the sample were boys, indicating that the validity of these tools for assessing persistent firesetting risk for girls and other genders has not been sufficiently investigated. Interestingly, the CRS and FRS tools were developed based on a sample of predominantly boys (95%) which is in line with much of the research indicating that more boys are engaged in firesetter interventions than girls.

The methods used to collect data in this study may also have influenced the findings. For example, following Fire‐CAP protocols, firesetters verbally responded to the questions on the CRS in the presence of their parents/carers, and may have felt uncomfortable fully disclosing information to a Fire‐CAP practitioner in front them. This could have caused them to alter their responses to avoid consequences or confrontation with their parents/carers after the session. This may explain why the FRS showed more promise as an effective triage tool than the CRS. However, the parent‐report method for collecting follow‐up information regarding continued firesetting behavior also has limitations regarding both knowledge of fire behavior, and a willingness to report.

Additionally, this study examined continued fireplay and/or firesetting within 12 months after the Fire‐CAP as reported by the parent/guardian. These behaviors were considered an overall category of persistent firesetting because the Fire‐CAP's goal is for children and adolescents to remain fire safe under all circumstances; and both of these acts, whether minimal or severe, involve a degree of risk. However, there may be differences that exist between “fireplayers” and “firesetters” with regard to the severity of their engagement. In future studies, it would be useful to consider the differences that may exist between firesetters in terms of the severity of their fires. Importantly, referral to mental health services does not guarantee the elimination of firesetting behavior. Notably, 60% of the persistent firesetters were in contact with mental health services. Knowledge of the treatment methods for firesetters used by practicing mental health professionals, and how mental health intervention is matched to risk needs is relatively limited, and should be explored further in the future.

## 9 IMPLICATIONS FOR PRACTICE AND FUTURE DIRECTIONS

Limitations aside, this is the first known published study to assess the predictive validity of the FRS and CRS for their ability to identify young people at‐risk of persistent firesetting. The FRS showed utility in effectively detecting persistent firesetters, but incorrectly classified nonpersistent firesetters as high risk over 50% of the time. The CRS was unable to effectively distinguish between persistent and nonpersistent firesetters. These findings highlight the potential problematic use of the CRS and FRS in their current standard form to inform treatment options for young firesetters. The use of the potentially flawed, over‐sensitive tools in practice means that a high proportion of young firesetters who could benefit from fire safety education delivered by fire services may be deemed ineligible. Given the current use of numerous old and under‐validated instruments to guide treatment in current firesetter intervention programs, it is critical to draw attention to potential flaws, areas for improvement, and the need for more thorough investigation into screening tools and any corresponding treatments. Future research should further explore the predictive validity of the CRS and FRS using a larger, representative sample of the population across different cultural contexts. Additionally, reconsideration of existing items included in the tools, and examining possible new items for the potential development of a new tool that suits the modern context and advances in knowledge since the inception of the CRS and FRS, should be undertaken. Research should also consider how firesetters are managed after risk is identified and the matching of intervention to risk, needs and responsivity. Thorough investigation into which components of interventions are most successful and for which firesetter is largely absent from the current literature (Johnston & Tyler, 2022). Together, these improvements will enable the triaging of persistent firesetters more effectively, and provide more meaningful interventions and pathways to treatment.

With young firesetter behavior being a clinical health issue with the potential to cause serious personal and social harm, it is imperative that fire services have effective screening processes and referral pathways. The findings here highlight the need for the development of an evidence‐based screening tool for young firesetter intervention programs to use.

## CONFLICT OF INTEREST

The authors declare no conflict of interest.

### PEER REVIEW

The peer review history for this article is available at https://publons.com/publon/10.1002/jclp.23435

## ETHICS STATEMENT

This study was approved by the Victoria University Human Research Ethics Committee.

## Supporting information

Supporting information.

## Data Availability

The data that support the findings of this study are available on request from the corresponding author. The data are not publicly available due to privacy or ethical restrictions.
